# Computed tomography angiography/magnetic resonance imaging-based preprocedural planning and guidance in the interventional treatment of structural heart disease

**DOI:** 10.3389/fcvm.2022.931959

**Published:** 2022-10-17

**Authors:** Dagmar Bertsche, Wolfgang Rottbauer, Volker Rasche, Dominik Buckert, Sinisa Markovic, Patrick Metze, Birgid Gonska, Erfei Luo, Tillman Dahme, Ina Vernikouskaya, Leonhard M. Schneider

**Affiliations:** Department of Internal Medicine II, Ulm University Medical Center, Ulm, Germany

**Keywords:** preprocedural planning, image fusion, structural heart disease, periprocedural guidance, annotated DICOM volume

## Abstract

Preprocedural planning and periprocedural guidance based on image fusion are widely established techniques supporting the interventional treatment of structural heart disease. However, these two techniques are typically used independently. Previous works have already demonstrated the benefits of integrating planning details into image fusion but are limited to a few applications and the availability of the proprietary tools used. We propose a vendor-independent approach to integrate planning details into periprocedural image fusion facilitating guidance during interventional treatment. In this work, we demonstrate the feasibility of integrating planning details derived from computer tomography and magnetic resonance imaging into periprocedural image fusion with open-source and commercially established tools. The integration of preprocedural planning details into periprocedural image fusion has the potential to support safe and efficient interventional treatment of structural heart disease.

## Introduction

An important prerequisite for successful interventional treatment of structural heart disease includes preprocedural planning (1–4), e.g., to determine the appropriate treatment option and to assess the target structure and accessible vessels, in most cases based on preprocedural non-invasive imaging (5–7). Furthermore, preprocedural estimation of device positioning has been shown to result in more efficient interventions (8–10).

Usually, preprocedural planning is performed by applying proprietary software systems (PSS), implemented either focusing on the preprocedural assessment of anatomical structures only [e.g., 3mensio Structural Heart™, Pie Medical Imaging, Maastricht, The Netherlands; Osirix™, Pixmeo Sàrl, Bernex, Switzerland; cvi42™, Circle Cardiovascular Imaging Inc., Calgary, AB, Canada; FluoroCT (11)] or focusing on intraprocedural image fusion with integrated planning modules as an add-on [e.g., EPNavigator™, HeartNavigator™, and EchoNavigator™, Philips Healthcare, Best, The Netherlands; syngo TrueFusion™ and Fusion Package™ (SHD), Siemens Healthineers, Erlangen, Germany; Valve ASSIST 2; GE Healthcare, Chicago, IL, USA]. As an alternative to proprietary software solutions for research and training purposes, BSD-style licensed open-source software tools (OSS) have been introduced for planning [e.g. 3DSlicer, www.3Dslicer.org (12)] and image fusion [e.g. 3DX-Guide (13)] of structural heart interventions. In addition to the assessment of the anatomy directly based on digital imaging and communications in medicine (DICOM) data, *in silico* implantation (14) and the use of 3D-printed models have recently been proposed both supporting preprocedural planning (15–17). During preprocedural planning, the identification of optimal fluoroscopic angulations has been reported to support the readily interpretation of patient-specific anatomy for improved navigation in fluoroscopy (18, 19). Fusion of contrast-enhanced computer tomography (CTA), echocardiography, or magnetic resonance imaging (MRI) with X-ray (XR) fluoroscopy improves guidance during complex catheter-based procedures (20–22). A reduction in procedural time and required contrast agent by periprocedural image fusion has also been reported for a variety of pre- and periprocedural imaging modalities (23, 24). Overlay of specific target locations in addition to the volumetric overlay of anatomical structures is effective (25), particularly regarding the fusion of echocardiography and fluoroscopy (26, 27).

The overlay of CTA-based planning generated with a planning-focused PSS onto XR fluoroscopy has been proposed for left atrial appendage occlusion as the future of image fusion without currently available software (28). The benefit of integrating planning details in image fusion for left atrial appendage occlusion has been demonstrated later using a specific PSS focused on image fusion (29, 30). Mainly in the context of transcatheter aortic valve replacement (TAVR), image fusion-focused PSS has been applied for integrated landmark determination and subsequent fusion with XR fluoroscopy (10).

In general, PSS planning modules integrated into software focusing on image fusion are user-friendly but lack compatibility with dedicated planning and/or image processing software to benefit from the respective advantage of different tools (31). There are free drawing tools included in PSS focused on image fusion to identify any anatomical abnormality and to assist in planning, but the tools often do not have the advantageous functionality that planning-focused software offers. Therefore, planning has to be done separately in both software, one for preprocedural assessment and planning and one for image fusion, which is time-consuming, error-prone, and less accurate than integrating the output of the planning-focused software into the image fusion-focused software.

It is the objective of the presented work to show the potential of the concept of fusing anatomical structures and planning data with XR fluoroscopy for different structural heart interventions using various combinations of software tools focusing on preprocedural planning or intraprocedural image fusion. CTA or MRI data have been used for preprocedural imaging and this approach has been tested with PSS and OSS as well as combinations of both to also demonstrate the applicability of this approach in a vendor-independent manner.

## Materials and methods

**FIGURE 1:**
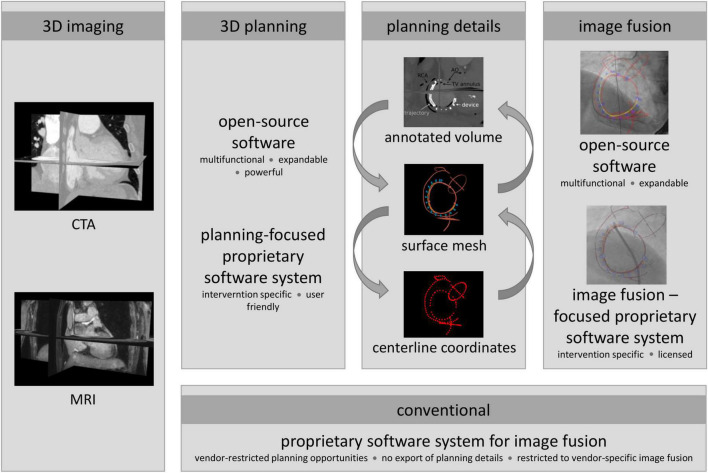
Proposed and conventional approach to include planning details during interventional procedures. Conventionally, a preprocedural 3D image is prepared for image fusion using an image fusion-focused proprietary software system (PSS), i.e., segmented and planning details added with integrated modules. Due to proprietary and vendor-specific implementation, data generated by independent sources, such as open-source software (OSS) or planning-focused PSS, can usually not be considered for intraprocedural guidance; however, providing more powerful planning support than fusion-focused PSS. To avoid the resulting restrictions, planning details exported from the planning tools as surface meshes, centerline coordinates, or annotated 3D DICOM image volumes were converted to generate an interface to the image fusion software. Thus, enabling the use of vendor-agnostic planning data during image fusion and procedural guidance.

Different combinations of PSS and OSS planning as well as image fusion software were used to exemplify the versatility and the resulting potential of including planning details in image fusion. The flexible approach of combining PSS and OSS in comparison to conventional vendor-specific solutions is schematically provided in Figure 1. Conventionally, planning details generated by OSS or planning-focused PSS cannot be considered during intraprocedural image fusion due to the non-compatibility of proprietary and vendor-specific interfaces. To avoid the resulting restrictions, an interface between vendor-specific and independent software packages for planning and image fusion was implemented based on the conversion of the planning data, enabling the use of vendor-independent data during image fusion and procedural guidance.

Based on patient-specific preprocedural 3D images, procedures were planned and relevant planning information was extracted using the PSS 3mensio™ or the OSS package 3DSlicer. Depending on the planning tool, planning details were exported as surface meshes, centerline coordinates, or annotated 3D DICOM image volumes. The PSS provides intervention-specific modules defining the export format either as centerline coordinates or as annotated 3D images. From centerline coordinates, a surface model was generated by placing vertices around each centerline coordinate with subsequent triangulation. This surface model can be used directly for image fusion with OSS. For use of image fusion-based PSS, an annotated image volume was generated by conversion of the surface model to a binary DICOM label map, which was merged with the preprocedural image volume. The resulting annotated volume is thus the preprocedural patient-specific 3D image in which the image voxel values of the identified planning details are marked by artificial intensities clearly contrasting from the surrounding anatomic intensities. The annotated volume is used as a workaround to enable the import of additional planning details into commercial PPS image fusion software. Moreover, planning details can be segmented from annotated image volumes using OSS or PSS similar to established surface models of anatomical structures. Using OSS for planning surface meshes were generated and either directly used for image fusion with OSS or converted to annotated 3D DICOM volumes for image fusion with PSS. For successful image fusion, the resulting surface models must be registered correctly with the XR system geometry. Initial registration was performed at the beginning of each procedure. The surface models were manually aligned in the XR coordinate system using two XR projections with an angular distance of at least 30° (ideal 90°) to ensure sufficient accuracy (32, 33). Whenever possible, manual alignment was performed on patient-specific (e.g., previously implanted artificial valves or ICD lead) or procedure-specific landmarks (e.g., catheters placed at the beginning of the intervention or contrast agent injection). In the absence of suitable landmarks, registration was based on the contour of the right atrium and aortic arch, which can normally be appreciated in the posterior-anterior and left-anterior-oblique XR projections. In all cases, registration was performed solely on data routinely acquired during the intervention with no additional contrast agent injection needed. Furthermore, projections routinely obtained during the intervention were used for on-the-fly refinement of the registration. Any changes in system geometry (angulation, table position, and zoom) were considered fully automated.

The suggested approach was applied exemplary for tricuspid annuloplasty, TAVR, transcatheter mitral valve replacement (TMVR), transseptal puncture (TSP), and left atrial appendage (LAA) occlusion. Image fusion of XR and CTA based-planning for tricuspid annuloplasty, TAVR, and TMVR as well as MRI-based planning of TSP were performed using the PSS EPNavigator™ and HeartNavigator™ (Philips Medical Systems B.V., Best, The Netherlands). Image fusion of MRI-based planning and XR for LAA occlusion was demonstrated postinterventionally using the XR-guidance OSS 3D-XGuide. For postinterventional image fusion, periprocedurally recorded DICOM sequences of the relevant steps during the intervention were exported from the XR system.

All pre- and periprocedural data used for the presented work have been acquired in full compliance with clinical guidelines as available. The image fusion of XR images and planning details was used as a confirmatory tool in conjunction with standard TEE and fluoroscopic techniques associated with the respective procedures. No formal guidance decisions were made solely from image fusion during the intervention. Patients provided written informed consent regarding the procedures and for the subsequent scientific use of the resulting imaging data before the intervention.

### Preprocedural imaging

Due to the high-spatial resolution of the imaging data and short acquisition time, CTA has preferably been used to provide preprocedural 3D anatomic data. CTA data were acquired with a SOMATOM Definition AS + (Siemens Healthineers, Erlangen, Germany) using the protocol previously reported for TAVR (34). 3D MRI data were acquired with 1.3^3^ mm^3^ resolution with a six-point mDixon sequence at 3 T (Achieva 3.0T, dStream, R5.6, Philips Medical Systems B.V., Best, The Netherlands) with a non-contrast-enhanced protocol as introduced by Homsi et al. (35).

### Intervention-specific application

#### Transcatheter tricuspid annuloplasty (15 cases, two interventional cardiologists)

**FIGURE 2:**
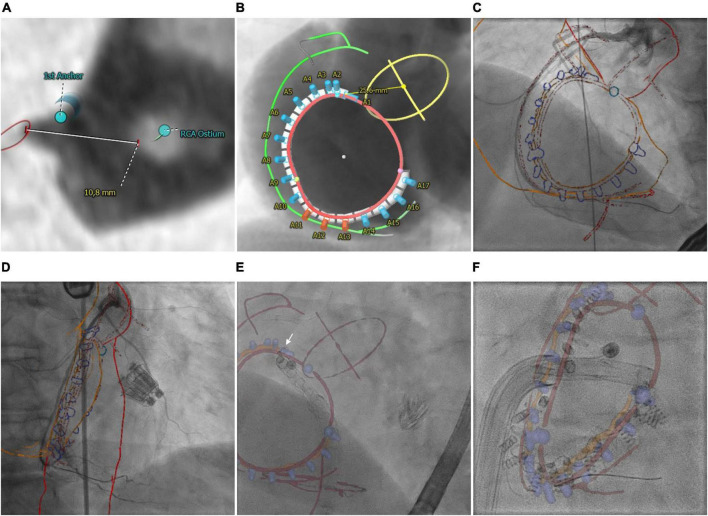
Transcatheter tricuspid annuloplasty. **(A)** CTA-derived optimal first anchor position. **(B)** Patient-specific anchoring is shown in angiographical simulation with marked anatomical landmarks including the right coronary artery (RCA, green), aortic root (AO, yellow), and tricuspid valve annulus (TV, red). **(C,D)** Manual registration based on angiographies of the RCA. **(E)** Image fusion of anatomical landmarks (red), trajectory (orange), and anchors (blue cylinders) during implantation of the first anchor (white arrow). The catheter is aligned with the vena cava inferior. **(F)** Image fusion of the lastly implanted anchor. Anchor heads are aligned with the planned trajectory. The commissures (blue spheres) indicated a potentially higher risk of the catheter slipping off the annulus.

According to previously reported recommendations (36, 37), anatomical landmarks, the target position of the Cardioband™ device (Edwards Lifesciences, Irvine, CA, USA) as well as a line connecting the anchor heads, called trajectory, were determined based on preprocedural CT using 3mensio™. Anatomical landmarks include the right coronary artery (RCA), tricuspid valve (TV) annulus and commissures, aortic annulus and center, the ostium of the coronary sinus, and course of the vena cava inferior. Anchoring and orientation of the Cardioband™ device around the annulus strongly depend on the patient-specific anatomy like distance to the center of the aortic root and RCA as well as tissue properties like thickness and contact area of the annular myocardium (Figures 2A,B). Especially, knowledge about regions of particular RCA proximity to the hinge point is crucial (Figure 2B, red markers). The TV module of 3mensio™ offers the possibility to export planning details included in the CTA volume on which planning is based. However, since planning details such as trajectory and anchors overlap, anatomical landmarks, device, and trajectory were exported as separate annotated image volumes. Subsequently, the three annotated image volumes were merged into one single image volume, including the annotated planning details in different grayscale voxel values using an if statement: if a voxel has been marked in one of the annotated image volumes, the corresponding voxel was also marked in the annotated volume with the planning detail specific grayscale value. The resulting annotated CTA volume was finally imported into HeartNavigator™ for semi-automatic segmentation. In addition to the planning details, the right atrium and aortic arch were segmented to enable registration. Registration was performed based on angiography of the RCA using a 30° left anterior oblique view (Figure 2C) and 30° right anterior oblique view (Figure 2D). To facilitate dynamic periprocedural image fusion, the HeartNavigator™ automatically updates the perspective of the overlay according to changes in XR system angulation.

#### Transcatheter aortic valve replacement (22 cases, two interventional cardiologists)

**FIGURE 3:**
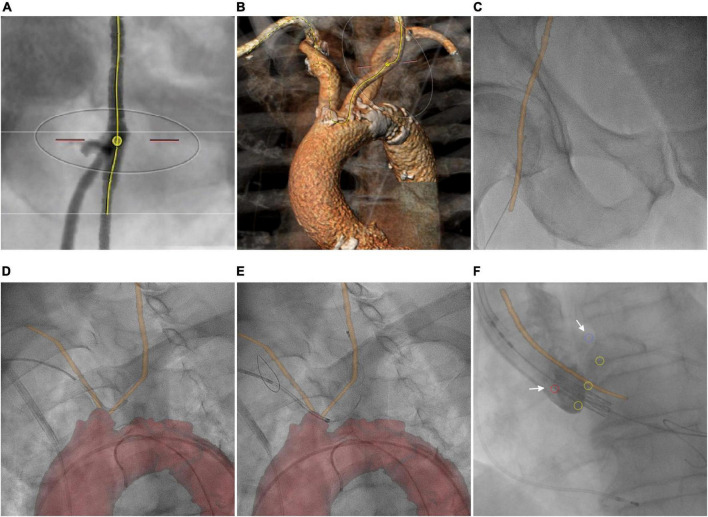
Transcatheter aortic valve replacement. **(A)** CTA-based determination of the transfemoral puncture site. **(B)** Volume rendering shows the route for implantation of the cerebral protection device. **(C)** Overlay of the femoral centerline on fluoroscopy. Image fusion of the patient-specific vascular centerline (orange line) during **(D)** and after **(E)** implantation of the cerebral protection system. **(F)** Depiction of aortic cusp hinge points (yellow circles), coronary ostia (red and blue circle, arrows), and aortic centerline (orange line) during valve implantation.

Besides the assessment of aortic root diameter and anatomy of ascending aorta and aortic valve for device selection and optimal positioning, preprocedural planning for TAVR includes evaluation of vascular access and route (38). Delineation of femoral, innominate, and carotid arteries helps to access puncture sites (Figure 3A) and facilitates device positioning for cerebral protection (Figure 3B; 39). Using 3mensio™ defined centerlines of the arteries are exported as point coordinates. Surface models and annotated CTA was generated from these coordinates. Using HeartNavigator™, the annotated CTA volume was segmented and coronary ostia and aortic cusps were marked. Initial registration of 3D anatomy to the XR system geometry was done based on pelvic structures with refinements performed based on the aortic arch.

#### Transcatheter mitral valve replacement (six cases, two interventional cardiologists)

**FIGURE 4:**
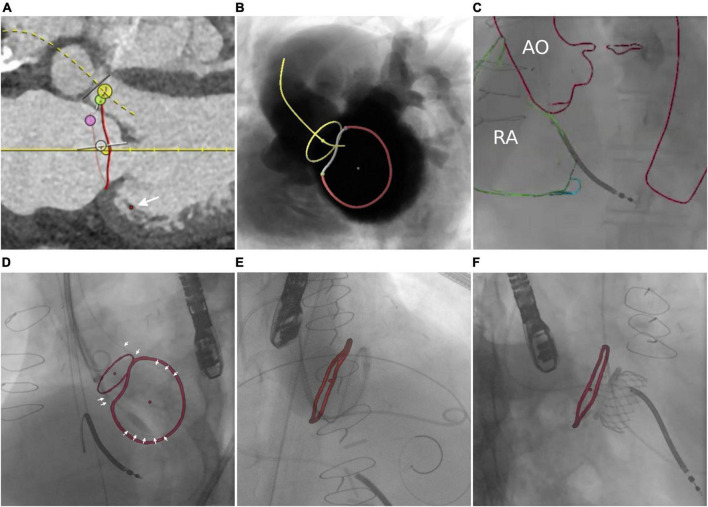
Transcatheter mitral valve replacement. **(A)** CTA-based identification of relevant anatomical landmarks, including pronounced trabecularization of subannular left ventricular walls (arrow) bearing the potential for entanglement during subannular looping. Determination of optimal C-arm angulation with panel **(B)** showing the simulated short-axis view including determined mitral (red) and aortic valve annuli (yellow). **(C)** Manual registration based on aorta (AO) and right atrium (RA). **(D)** Image fusion in the short axis view shows the loop placement catheter (white arrows) aligned to the mitral valve annulus. Image fusion in a 4-chamber view indicates the target area and correct positioning of the ring to which the prosthesis is attached **(E)** and a good correlation between successfully implanted HighLife™ valve after valve implantation **(F)**.

Based on preinterventional CTA mitral and aortic valve annuli were identified as previously recommended (40). Different from other transcatheter mitral valve replacement systems like Intrepid™ (Medtronic plc, Dublin, Ireland) or EVOQUE Eos™ (Edwards Lifesciences, Irvine, CA, USA), the HighLife™ valve (HighLife SAS, Paris, France) uses a subannular ring as landing zone similar to the Sapien M3™ valve (Edwards Lifesciences, Irvine, CA, USA) (41, 42). Therefore, pronounced trabecularization of subannular left ventricular walls (arrow) or subvalvular apparatus bearing the potential for entanglement of the wire used for subannular looping were depicted (Figure 4A). Finally, optimal XR angulations of annular planes according to Piazza et al. (18) were determined to facilitate subannular wire looping (Figure 4B shows the simulated short-axis view) as well as optimal device positioning. Using 3mensio™, the planning details were exported as annotated CTA volume, which was further processed using EPNavigator™ to generate surface models and perform image fusion. Manual registration was done by alignment of volumetric segmentation of the right atrium (RA) and aorta (AO) to their XR projections (Figure 4C).

#### Transseptal puncture (17 cases, three interventional cardiologists)

**FIGURE 5:**
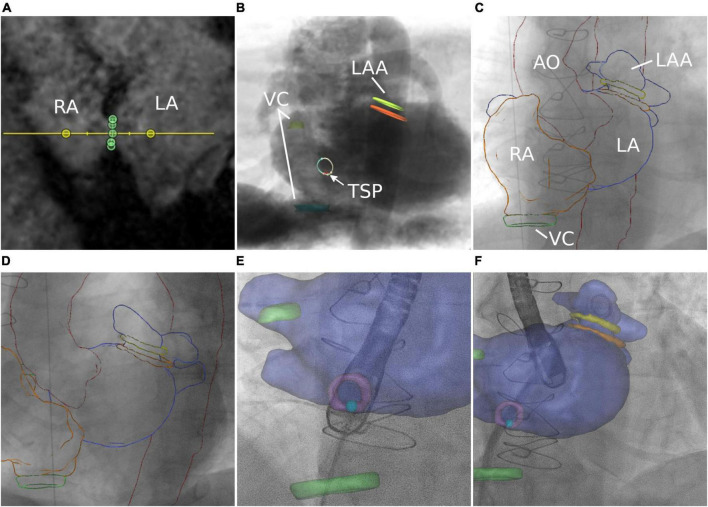
Transseptal puncture. **(A)** MRI-based identification of the interatrial septum. **(B)** Simulation of posterior–anterior projection including MRI-based identification of left atrial appendage (LAA), septum, transseptal puncture (TSP), and vena cava (VC). **(C,D)** Manual registration is based on the aortic arch (AO) and right atrium (RA). Image fusion during TSP **(E)** and after successful access to the target structure **(F)**.

The interatrial septum was identified based on preprocedural MRI. Figure 5A shows the MRI-derived septal plane between the left (LA) and right atrium (RA). The desired location of TSP was defined using 3mensio™ according to patient-specific anatomy, relation to vena cava (VC) inferior, and type of intervention as previously reported (43)—in this case, LAA occlusion (Figure 5B). Using EPNavigator™, a 3D marker, was set on the segmented LA as previously determined targeting the desired TSP. XR projections of RA (orange outline) and aorta (AO, red outline) were used for registration in posterior-anterior (Figure 5C) and 40° left anterior oblique orientation (Figure 5D).

#### Left atrial appendage occlusion (28 cases, three interventional cardiologists)

**FIGURE 6:**
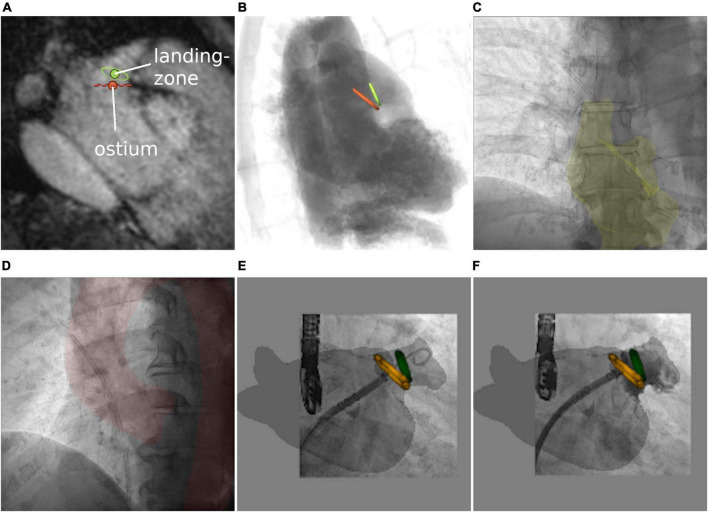
Left atrial appendage occlusion. **(A)** MRI-based assessment of left atrial appendage (LAA) shape and diameters. **(B)** Angiographical simulation including defined ostium (orange) and landing zone (green). **(C,D)** Manual registration based on right atrium and aortic arch. Image fusion includes planning details during the approach to the LAA **(E)** and after occlusion **(F)**.

As previously reported for CTA data (44), the shape and diameter of the LAA (orange—ostium, green—landing zone) were assessed using preprocedural 3D MRI, enabling the selection of the most suitable occlusion device (Figure 6A). Based on simulated angiography, the optimal XR angulation for perpendicular implantation with regards to ostium and landing zone was determined (Figure 6B). 3mensio™ planning details were exported as annotated DICOM volume. Segmentations were generated using 3DSlicer. A retrospective image fusion was performed with 3D-XGuide. Registration was performed with respect to the RA (yellow overlay) in posterior–anterior projection (Figure 6C) and aortic arch (red overlay) in 40° left anterior oblique view (Figure 6D). If necessary, registration was improved during the intervention based on the catheter placed in the left upper pulmonary vein.

## Results

Due to the flexible conversion of preprocedural planning and anatomical surface models, their integration was successful in all cases. Delineation of anatomical structures and planning information could be derived for all interventions, as mentioned in the methods section. Although the resulting accuracy of the registration could not be quantified and was still limited by not considering the cardiac and respiratory motion, there was a general agreement between the physicians on improved anatomical reference during procedures with the use of fusion imaging. For all interventions, the fusion approach in its current form was already appreciated as a promising adjunct to the established XR and transesophageal echocardiography (TEE) guidance. In the following, the specific intervention-related assessments are listed explicitly.

### Transcatheter tricuspid annuloplasty

Overlay of planning details with fluoroscopy particularly helped to guide the catheter into the target region of the first anchor (arrow, Figure 2E and Supplementary Video 1). Even if the exact anchor positions deviated from the planned anchor positions (blue), the trajectory (orange) enabled the estimation of the targeted distance between RCA and annulus (red) (Figure 2F). Moreover, the planned anchor positioning/orientation could be used to align the catheter prior to final positioning under TEE guidance.

### Transcatheter aortic valve replacement

Periprocedural registration of 3D anatomy to the XR system geometry based on pelvic structures enabled the overlay of targeted puncture sites (Figure 3C). During implantation of the cerebral protection system (SENTINEL™, Boston Scientific Corporation, Marlborough, MA, USA), overlay of the specific vascular centerline supported catheter guidance and device positioning (Figures 3D,E). Finally, the depiction of aortic cusp hinge points, coronary ostia, and aortic centerline supported the correct trajectory and positioning of the prosthesis (Sapien3™, Edwards Lifesciences, Irvine, CA, USA) (Figure 3F).

### Transcatheter mitral valve replacement

Overlaying planning details particularly supported guidance of the loop placement catheter in relation to mitral and aortic valve annular planes and as such may help to avoid entanglement with trabecular structures of the left ventricle and subvalvular apparatus (Figure 4D and Supplementary Videos 2, 3). Eventually, the overlay of the mitral valve annulus allowed verification of correct subannular wire looping as well as positioning of the ring to which the prosthesis is being attached (Figure 4E). Moreover, the annular plane of the mitral valve overlaid on XR indicates the target area for valve positioning. Figure 4F shows a good correlation between the successfully implanted HighLife™ valve and the previously determined mitral annulus.

### Transseptal puncture

Image fusion supported the placement of the catheter toward the target area (light blue point) within the septum (purple circle). Preprocedural planning of TSP location was used for intraprocedural guidance by transesophageal echocardiography (Figure 5E and Supplementary Video 4). Optimal puncture location facilitated optimal access to the target structure (Figure 5F).

### Left atrial appendage occlusion

Volumetric overlay particularly supported understanding of the LAA shape including preprocedural defined ostium and landing zone (Figure 6E) and provided intraprocedural control of the device location after deployment [Figure 6F shows an implanted occluder (Watchman FLX™, Boston Scientific Corporation, Marlborough, MA, USA) at the exact position of the predicted landing zone (Supplementary Video 5)].

## Discussion

First of all, it has to be mentioned explicitly that the use of fusion software is not yet applicable to replace established guidance approaches based on XR or TEE. As already discussed by Kliger et al. (45), major limitations regarding accuracy rise from a still imperfect registration between preprocedural and periprocedural data. This is mainly caused by not considering cardiac and respiratory motion and anatomical mismatches between the pre- and periprocedural circumstances like different patient positioning or volume status. However, even in its current form, the fusion approach was reported to have the potential to greatly support the established procedures as an adjunct to XR and TEE imaging. Especially, the additional anatomical overview and provided planning details were well-received potentially improving the efficiency and safety of the investigated procedures in the future.

### Key findings

This work demonstrates the feasibility of integrating planning details into image fusion in PSS and OSS using annotated DICOM volumes. Where current image fusion-focused technology is limited to integrated planning modules with restricted functionalities and vendor-specific hardware, utilizing planning-focused PSS offers semiautomatic tools for accurate assessment and planning. The combination of both enables intraprocedural image fusion of XR with accurate planning details generated once during the assessment. OSS can be used to explore new research approaches or overcome limitations of the PSS and enable vendor-agnostic interfaces by applying standardized formats. Besides the exemplarily shown combinations of OSS and PSS-based planning and image fusion software, further combinations are possible. Even though in the presented applications a combination of PSS and OSS was used, sole use of OSS is feasible as such enabling rapid evaluation of new algorithms and techniques. Whereas, commercially approved image fusion tools provide support for periprocedural imaging, open-source tools facilitate the elaboration of research approaches or retrospective analysis. Typically, PSS is implemented in a use-case-specific manner and does not allow any or only very restricted interfaces to other software solutions. Contrarily, OSS is often multifunctional but less user-friendly. Utilizing the DICOM standard eliminates the need for an additional interface and thus enables the exchange of planning details between the various software solutions. With this approach, guidance during interventional treatment is supported by image fusion including patient-specific planning details independent of the type of intervention and software tools. Since preprocedural planning is often already done for the preparation of the procedure, the additional effort for integrating planning details into the image fusion is rather low.

Importantly, we could demonstrate the feasibility of using MRI-derived planning details as a radiation-free alternative to CTA-based planning.

Although not yet widely used for procedural guidance, the presented applications indicate a potential benefit in integrating preprocedural planning details into periprocedural image fusion for the treatment of different structural heart diseases. In all cases, the integration of detailed anatomical landmarks (e.g., annular and septal planes) and planning details (e.g., Cardioband™ anchor positions or Watchman FLX™ landing zones) was considered helpful by the interventional cardiologists involved in addition to regular use of fluoroscopy and echocardiography. Such complementary information promotes quick and precise orientation even in complex anatomies and may increase patient safety as well as reduce procedure time and anesthesia in the future.

### Limitations

A major limitation of the presented approach is the non-certified use of PSS or OSS. As such, the fused data was not used to draw any decisions during the intervention, and the assessment of the added value could not be quantified but relied on the subjective impression of the treating physicians. Even though there is currently only subjective evidence that the proposed approach might improve the efficiency and safety of certain interventional procedures, it might be suitable for training, retrospective analysis, or research already at the current stage.

Furthermore, the accuracy of image fusion is limited. Registration accuracy is limited by differences between the pre- and periprocedural circumstances, such as patient positioning and hemodynamic condition. Whereas, all system parameters are automatically considered for updating the established registration, the patient motion may demand readjustment of the registration or even re-registration. Moreover, the use of static surface models does not yet allow for a complete consideration of the cardiac and respiratory motion.

However, despite the limitations mentioned above regarding accuracy, the fusion of preprocedural data and live fluoroscopy supports periprocedural navigation (46).

### Future considerations

For full utilization of the potential of the proposed technique, motion compensation and automatic registration are required. In addition to using CTA or MRI for planning, alternative imaging modalities such as echocardiography might be of interest as well as a fusion of XR alternatives to periprocedural imaging like TEE or intracardiac echocardiography.

## Conclusion

The feasibility and potential benefit of various combinations of planning-focused and image fusion-focused proprietary and open-source software solutions were demonstrated for different structural heart interventions. The concept of fusing preprocedural 3D-image-based planning details derived from planning-focused software with live XR fluoroscopy shows great potential to support efficient and secure periprocedural guidance in the interventional treatment of structural heart disease. However, the use of OSS is limited to research and training, as it is not approved as a medical device. Furthermore, limitations regarding accuracy due to static overlay and manual registration of 3D planning to the XR system geometry may need further attention.

## Data availability statement

The original contributions presented in this study are included in the article/Supplementary material, further inquiries can be directed to the corresponding author.

## Ethics statement

The studies involving human participants were reviewed and approved by Ethikkommission der Universität Ulm. The patients/participants provided their written informed consent to participate in this study.

## Author contributions

DBu and BG: study setup, performance, and contribution to data interpretation of TAVR. WR, LS, and SM: study setup, performance, and contribution to data interpretation of TMVR. TD, LS, and BG: study setup, performance, and contribution to data interpretation of LAA and TSP. LS and SM: study setup, performance, and contribution to data interpretation of transcatheter annuloplasty. PM and EL: optimization of MRI sequence. DBe: conception and design of the study, the performance of image fusion, data collection and analysis, and manuscript drafting. LS, IV, and VR: critical revision for intellectual content. All authors have read and approved the final manuscript.
